# The Pathological Spectrum of Systemic Anaplastic Large Cell Lymphoma (ALCL)

**DOI:** 10.3390/cancers10040107

**Published:** 2018-04-04

**Authors:** Ivonne A. Montes-Mojarro, Julia Steinhilber, Irina Bonzheim, Leticia Quintanilla-Martinez, Falko Fend

**Affiliations:** Institute of Pathology and Neuropathology and Comprehensive Cancer Center Tübingen, Eberhard-Karls-University, Liebermeisterstraße 8, 72076 Tübingen, Germany; Ivonne.Montes@med.uni-tuebingen.de (I.A.M.-M.); Julia.Steinhilber@med.uni-tuebingen.de (J.S.); Irina.Bonzheim@med.uni-tuebingen.de (I.B.)

**Keywords:** anaplastic large cell lymphoma (ALCL), ALK (anaplastic lymphoma kinase), breast implant-associated ALCL, lymphoma, morphology

## Abstract

Anaplastic large cell lymphoma (ALCL) represents a group of malignant T-cell lymphoproliferations that share morphological and immunophenotypical features, namely strong CD30 expression and variable loss of T-cell markers, but differ in clinical presentation and prognosis. The recognition of anaplastic lymphoma kinase (ALK) fusion proteins as a result of chromosomal translocations or inversions was the starting point for the distinction of different subgroups of ALCL. According to their distinct clinical settings and molecular findings, the 2016 revised World Health Organization (WHO) classification recognizes four different entities: systemic ALK-positive ALCL (ALK+ ALCL), systemic ALK-negative ALCL (ALK− ALCL), primary cutaneous ALCL (pC-ALCL), and breast implant-associated ALCL (BI-ALCL), the latter included as a provisional entity. ALK is rearranged in approximately 80% of systemic ALCL cases with one of its partner genes, most commonly *NPM1*, and is associated with favorable prognosis, whereas systemic ALK− ALCL shows heterogeneous clinical, phenotypical, and genetic features, underlining the different oncogenesis between these two entities. Recognition of the pathological spectrum of ALCL is crucial to understand its pathogenesis and its boundaries with other entities. In this review, we will focus on the morphological, immunophenotypical, and molecular features of systemic ALK+ and ALK− ALCL. In addition, BI-ALCL will be discussed.

## 1. Introduction

Anaplastic large cell lymphoma (ALCL) represents a group of malignant lymphoproliferations sharing morphological and immunophenotypic features, but exhibiting different clinical and genetic characteristics [1]. The 2016 revised World Health Organization (WHO) lymphoma classification recognizes four different entities: systemic ALK-positive ALCL (ALK+ ALCL), systemic ALK-negative ALCL (ALK− ALCL), primary cutaneous ALCL (pC-ALCL), and the provisional entity breast implant-associated ALCL (BI-ALCL) [1].

ALCL was recognized for the first time by Stein et al. in 1985 as “anaplastic large cell Ki-1-positive lymphomas”; an aggressive non-Hodgkin’s lymphoma (NHL) exhibiting a morphology suggestive of “malignant histiocytosis” (MH), but with immunohistochemical features overlapping with those of classic Hodgkin’s lymphoma (CHL). This entity was defined by a proliferation of large lymphoid cells with a cohesive growth pattern with a tendency to invade lymph node sinuses and strong expression of Ki-1 antigen (later designated CD30) [2]. Subsequently, ALCL was recognized as a distinctive entity and incorporated into the Kiel classification in 1988, the Revised European–American Lymphoma (REAL) classification in 1994, and finally into the WHO classification of haematopoietic and lymphoid tissues in 2001 [3,4]. Although the original descriptions of this entity considered ALCL a neoplasm related to CHL and MH, subsequent immunophenotypic and T-cell receptor (TCR) gene rearrangement studies confirmed its derivation from T cells and led to the recognition of ALCL as a unique subtype of peripheral T-cell lymphoma (PTCL) lacking expression of T-cell related surface proteins [2,5,6]. In 1994, the discovery of the recurrent t(2;5)(p23;q35) translocation fusing the anaplastic lymphoma kinase (*ALK*) gene and the nucleophosmin (*NPM1*) gene in the majority of, but not all, cases of ALCL indicated that this entity contained at least two distinct subtypes [7]. Whereas ALK+ ALCL was recognized as a definite entity already in the 2008 WHO classification, systemic ALK− ALCL remained as a provisional entity, due to the lack of clear-cut criteria to distinguish it from other CD30-positive PTCLs [8]. However, the differences in epidemiology, clinical outcomes, and recently, novel molecular findings have supported its distinction from other PTCL [9,10], and nowadays ALK− ALCL is recognized as a distinct entity [1]. Another subgroup of ALCL associated with the seroma forming around breast implants has been added as a new provisional entity, designated breast implant-associated ALCL (BI-ALCL) [1]. The etiology and pathogenesis of BI-ALCL have been related to the immune reaction to silicone, and there are currently no known recurrent genetic aberrations [11]. Further studies are needed in order to learn about its pathogenesis [12].

The main focus of this review will be on the morphological, immunophenotypical, and genetic features of systemic ALCL. In addition, the new insights about BI-ALCL will be discussed (Table 1).

## 2. Systemic ALK-Positive Anaplastic Large Cell Lymphoma (ALK+ ALCL)

### 2.1. Definition and Clinical Features

ALK+ ALCL is a type of PTCL consisting of large lymphoid cells with abundant cytoplasm and pleomorphic, often horseshoe-shaped nuclei, characterized by strong CD30 immunostaining and ALK chromosomal translocation [1]. It accounts for 1–3% of adult and 10–20% of pediatric and adolescent NHL [1,13]. Systemic ALK+ ALCL predominantly occurs in children and young adults with a slight male predominance. It shows an aggressive behavior with rapidly progressive adenopathy and systemic symptoms such as fevers, night sweats, and weight loss. At the time of diagnosis, most patients are in an advanced stage of disease (III–IV stage) with systemic symptoms (75%) and lymph node enlargement (90%), including mediastinal involvement (36%). Extranodal involvement is present in 40–68% of cases, including skin (26%), bone (14%), and soft tissues (15%), lung (12%), and liver (8%) [14,15,16,17]. Central nervous system involvement is rare; patients are usually younger at onset and are considered to have a high-risk disease, but still can achieve complete remission after chemoradiotherapy in a significant percentage of cases [18,19,20]. Leukemic involvement is rare and prognostically unfavorable and most commonly occurs in the small cell variant of ALK+ ALCL [21,22]. Another interesting clinical feature is the presence of circulating antibodies against ALK fusion protein at time of diagnosis and during remission of ALK+ ALCL [23,24]. Titers of ALK autoantibodies correlate with clinical stage, and low autoantibodies titers are usually associated with a significantly higher incidence of relapses [25]. Bone marrow (BM) involvement is observed in up to 40% of the cases when immunohistochemical analysis is performed [26].

### 2.2. Morphological Features

**Figure 1:**
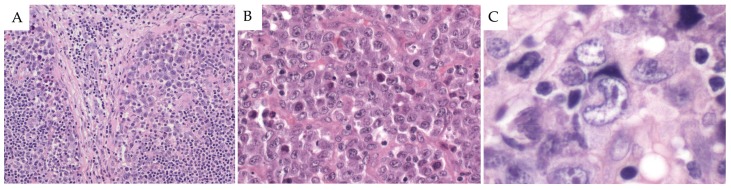

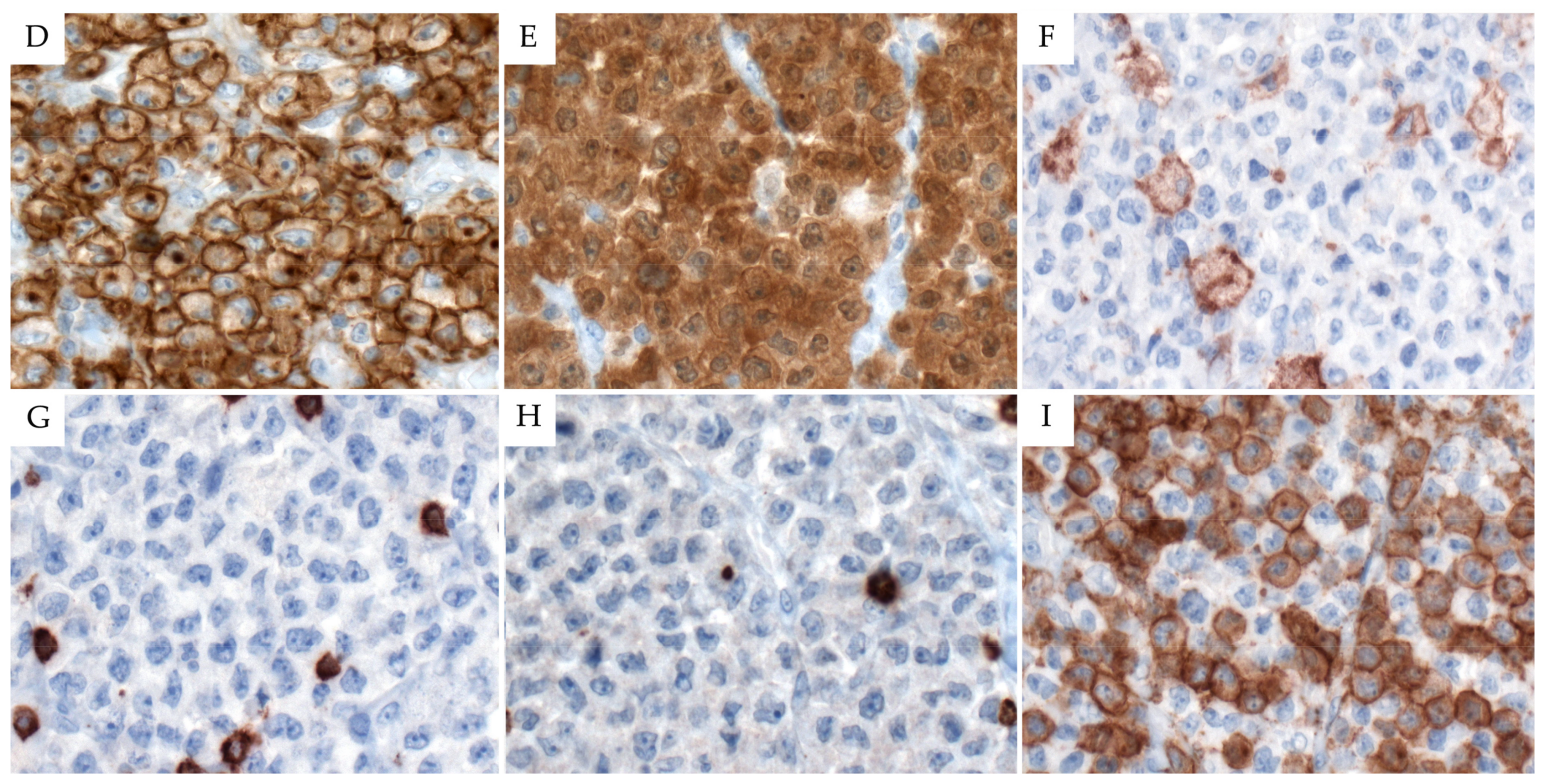
Morphological and immunohistochemical findings in ALK+ ALCL. (**A**) Tumor cells infiltrating the sinuses displaying a cohesive pattern (H&E stain, 100×). (**B**) The large neoplastic cells are relatively monomorphic, showing abundant eosinophilic cytoplasm and pleomorphic nuclei and frequent apoptotic bodies (H&E stain, 400×). (**C**) A “hallmark cell”, displaying an eccentric horseshoe-shaped nuclei with two nucleoli and prominent Golgi area (H&E stain, 630×). (**D**) The tumor cells are strongly and uniformly positive for CD30 with a membranous and Golgi zone pattern. (**E**) Strong cytoplasmic nuclear and nucleolar ALK staining in the neoplastic cells is observed. (**F**) CD4 is positive in macrophages but negative in neoplastic cells. (**G**,**H**) CD3 and CD7 are positive in accompanying T cells but negative in the tumor cells. (**I**) CD5 is positive in the majority of the tumor cells ((**D**–**I**), immunohistochemistry, 400×). Abbreviations: H&E: hematoxylin and eosin.

ALK+ ALCL shows a significant morphological heterogeneity, ranging from small to medium-sized cells to cases where anaplastic large cells predominate. Usually the architecture of lymph nodes is partially effaced in ALK+ ALCL; the tumor cells commonly infiltrate the subcapsular sinuses and paracortical region, often showing a striking intrasinusoidal dissemination in a sheet-like pattern, mimicking a metastatic carcinoma (Figure 1A) [15,27,28,29]. The characteristic neoplastic cells of this lymphoma are large with abundant eosinophilic cytoplasm and prominent Golgi apparatus, stained as a clear perinuclear zone. Nuclei are large and show open chromatin with multiple nucleoli. Neoplastic cells with eccentric, horseshoe, or kidney-shaped nuclei have been referred to as “hallmark cells”, (Figure 1C), because they are present in all morphological variants of this lymphoma, and therefore constitute a characteristic morphological feature [28,30]. Routine morphological examination of BM biopsies reveals involvement in approximately 15% of the cases; the frequency of involvement is usually underestimated due to the difficulty of distinguishing isolated neoplastic cells from other BM cells. The use of immunohistochemical markers such as CD30, epithelial membrane antigen (EMA), and ALK has increased the detection rate to 40%, highlighting the hidden tumor cells [26]. Using reverse transcription polymerase chain reaction (RT-PCR) for the detection of NPM-ALK transcripts, the positivity rate of BM aspirates is even higher and may have prognostic significance [31]. Rare cases with BM involvement associated with extreme neutrophilia as a result of increased IL-17 secretion have been reported and are associated with an aggressive clinical course [32].

According to the cytological and architectural features, five patterns have been recognized in the WHO classification: the common pattern, the small cell pattern, the lymphohistiocytic pattern, the Hodgkin’s-like pattern, and the composite pattern [1]. These different histological patterns are not considered histological grades, since overall they show a similar favorable prognosis [30]; however, it is important to recognize the small cell and the lymphohistiocytic patterns because they have been associated with more frequent leukemic manifestation and other features conveying a more aggressive course [22,33,34].

#### 2.2.1. Common Pattern

The ALK+ ALCL common pattern represents the most frequent morphological variant (60–70%). It consists predominantly of large pleomorphic cells with admixed “hallmark” cells: they have abundant clear or basophilic cytoplasm with large pleomorphic nuclei, finely dispersed nuclear chromatin, and multiple small nucleoli [27,29]. Occasionally, cells with multiple nuclei, sometimes arranged in a wreath-like fashion resembling Reed–Sternberg (RS) cells are found, but neoplastic cells usually lack the large viral inclusion-like nucleoli of the characteristic RS cells. In addition to hallmark cells, it is common to find other large pleomorphic cells that seem to have nuclear inclusions referred to as “doughnut cells”; these inclusions are section artefacts, representing only invaginations of the nuclear membrane [27]. Another characteristic feature is the invasion of the lymph node sinuses accompanied by perivascular distribution. This distribution can also be seen in the other morphological variants of ALCL. CD30 immunostaining is useful to identify the neoplastic cells in cases with subtle sinusoidal involvement, especially in the small cell and in the lymphohistiocytic variants [27].

#### 2.2.2. Small Cell Pattern

The small cell pattern (5–10%) shows a predominant population of small to medium-sized neoplastic cells with clear cytoplasm and distinctive cell membranes exhibiting a “fried egg” appearance. The nucleus of the neoplastic cells can be horseshoe-shaped or round, and typical hallmark cells are always present and concentrated around the blood vessels, forming rosettes. This pattern can be easily misdiagnosed as a PTCL “not otherwise specified” (PTCL-NOS), which can display the same morphology accompanied by variable CD30 positive immunostaining [30,35].

#### 2.2.3. Lymphohistiocytic Pattern

The lymphohistiocytic pattern (10%) is characterized morphologically by the presence of small neoplastic cells admixed with abundant histiocytes. The histiocytes can predominate in this pattern, masking the neoplastic cells, making the diagnosis of a malignancy challenging without appropriate immunostaining. Occasionally, the histiocytes show signs of erythrophagocytosis and a monomorphic appearance, which has led to a misdiagnosis of this entity as MH in the past [36]. Based on the fact that small cell and lymphohistiocytic patterns can appear alone or intermixed in the same tumor or in subsequent biopsies, these two patterns seem to be closely related [27]. Commonly, neoplastic CD30-positive cells are found also in the peripheral blood, representing a leukemic manifestation that is particular of these two patterns and is usually not observed in the other morphological variants [22,34].

#### 2.2.4. Hodgkin’s-Like Pattern

The Hodgkin’s like pattern is present in only 3% of ALK+ ALCL. The morphological features include an architecture that resembles nodular sclerosis CHL with a prominent inflammatory background [37,38]. ALK immunostaining is crucial in the differential diagnosis of this entity; cases will be misdiagnosed as CHL when ALK+ ALCL is not considered and ALK immunostaining is not performed.

#### 2.2.5. Composite Pattern

In about 15% of the cases, more than one pattern can be seen in a lymph node biopsy, referred to as a “composite pattern” [27]. Other rare morphological patterns such as the sarcomatous variant also have been described, but they are not integrated as a specific morphological pattern in the WHO classification [39]. Cases with tumor cells distributed in a myxoid background may be misdiagnosed as soft tissue sarcomas.

### 2.3. Immunophenotype

Immunophenotypically, ALK+ ALCL shows strong and uniform expression of CD30, a member of the tumor necrosis factor receptor (TNFR) superfamily [2,40]. However, CD30 expression is not specific for ALCL tumor cells, and is also found in activated B or T cells [41,42], in some solid tumors [43,44,45], in CHL [2], as well as in a subset of PTCL-NOS [46]. CD30 is expressed strongly and homogenously on the cell membrane and in the Golgi region (Figure 1D) in all the morphological patterns, except for cases of small cell variant ALCL [15].

In 1997, Pulford developed a monoclonal antibody for paraffin sections, which detects ALK protein expression with a high sensitivity and specificity [47]. Since the ALK protein is not expressed outside the central nervous system in normal tissues postnatally, specific detection of ALK protein confirms a malignant neoplasm, but not necessarily ALK+ ALCL, since other tumors with ALK rearrangements exist; for example, a subset of adenocarcinoma of the lung. The immunostaining for ALK protein shows a strong correlation with the presence of rearrangements detected by other genetic study methods such as karyotyping, fluorescence in situ hybridization (FISH), or sequencing; therefore, ALK immunohistochemistry became the gold standard for the diagnosis of ALK+ ALCL. According to the *ALK* translocation partner gene, ALK immunohistochemistry shows different patterns of staining, which is strongly dependent on the *ALK* binding partner. Cases with *NPM1-ALK* translocation show cytoplasmic, nuclear, and nucleolar staining. The cytoplasmic staining is the result of the link between the intracytoplasmic portion of ALK and the N-terminal portion of NPM1, whereas nuclear and nucleolar staining are explained by the colocalization the of NPM1-ALK fusion protein to the nucleus through the formation of heterodimers between wild-type *NPM1* and *NPM1-ALK* [47]. In the small cell variant, ALK staining is usually confined to the nucleus. *ALK* variant translocation partners such as *TPM3*, *ATIC*, *TFG*, *CLTC*, *TPM4*, and *MYH 9* lack nuclear localization and the staining pattern is limited to the cytoplasm. However, *MSN–ALK* translocation shows a unique membrane staining that is thought to be the association of myosin with cell membrane proteins [48,49,50]. Most of the cases of ALK+ ALCL express one or more T-cell antigens, but the loss of several pan-T-cell antigens is commonly observed, resulting in a so-called null-cell phenotype (Figure 1F–H) [51]. Although neoplastic cells frequently lose several T-cell antigens, the T-cell origin is supported by the presence of TCR rearrangements in 80% to 90% of cases and gene expression profiling [6,51]. Neoplastic cells are variably positive for CD45 and CD45RO, but negative for CD3 in more than 75% of the cases. In contrast, other T-cell antigens such as CD2, CD5, and CD4 are positive in variable subsets of cases (40–70%). Although CD8 is usually negative, most of the cases express cytotoxic associated antigens such as TIA-1, granzyme B, and perforin (Table 2) [9]. TCR betaF1 antibody is only expressed in 4% of the cases. Interestingly, the loss of expression of TCR-associated molecules (CD3, ZAP70, LAT, and SLP76) is usually accompanied by strong positivity of CD25 (IL-2 receptor), an antigen that has been related to epigenetic downregulation of the TCR signaling pathway [52]. CD15 and PAX5 expression is rare and usually restricted to a minority of cells, but when these markers are expressed in ALCL, this may lead to diagnostic confusion with CHL. In those cases, CD45 and EMA are useful markers, which are frequently positive in ALK+ ALCL and usually negative in CHL. The transcription factor IRF4/MUM1 and fascin, a protein involved in the formation of dendritic processes, are both strongly positive in RS cells, but also expressed in ALCL cells; therefore, not useful for the differential diagnosis [53,54,55]. The extracellular chaperone clusterin is highly expressed in systemic ALCL, showing a characteristic Golgi staining pattern; however, clusterin reactivity has also been shown in a subset of diffuse large B-cell lymphomas (DLBCL), and in cases of PTCL-NOS and CHL [56]. A minority of cases of ALK+ ALCL express the natural killer cell antigen CD56 [57]. Other markers highly expressed in ALCL which are not used for practical diagnosis, but play an important role in its oncogenesis are SHP-1 phosphatase, BCL6, C/EBPβ, CD147, and CD44v6 [58,59,60,61,62,63]. Interestingly, 50% of ALK+ ALCL cases analyzed by tissue microarrays are positive for SHP-1, a negative regulator of the NPM-ALK signaling pathway, which plays an important role in the oncogenesis and has become a therapeutic target in this lymphoma [58]. Epstein-Barr virus (EBV) is consistently negative in ALK+ ALCL [1]. In general, the immunophenotype including the variant expression patterns of ALK are not associated with specific prognostic features. Immunohistochemical markers suggested to be related to prognosis are SERPIN A-1 and survivin. The modulation of these markers may provide a novel target for experimental therapy in patients with systemic ALCL [57,59,64].

### 2.4. Genetic and Molecular Findings

Approximately 80% of the cases show a cytogenetic translocation t(2;5), which fuses the *ALK* gene, a receptor tyrosine kinase domain, at 2p23 to the nucleophosmine (*NPM*) gene at 5q35, resulting in the overexpression and constitutive activation of a chimeric ALK fusion protein, which plays an important role in ALK-mediated oncogenesis [7]. Other ALK variant translocations also occur (Table 3), involving tropomyosin 3 (*TPM3*: 1q25) in 13% of cases; 5-aminoimidazole-4-carboxamide ribonucleotide formyltransferase/IMP cyclohydrolase (*ATIC*: 2q35) in 1%; TRK-fused gene (*TFG*: 3q12.2) in 1%; and in <1% of cases, clathrin heavy chain (*CLTC*: 17q23), moesin (*MSN*: Xq11-12), tropomyosin 4 (*TPM4*: 19p13.1), myosin heavy chain 9 (*MYH9*: 22q11.2), ring finger protein 213 (*RNF213*: 17q25), and TNF receptor-associated factor 1 (*TRAF-1*: 9q33.2) [48,49,50,68,69,70,71,72,73,74,75,76,77]. All the variant translocations lead to the constitutive activation of ALK-tyrosine kinase and represent the critical alteration in this lymphoma [78,79].

The ALK portion of the NPM-ALK protein containing the catalytic domain of the ALK receptor undergoes autophosphorylation through reciprocal ALK tyrosine kinase activity, leading to strong and persistent activation [80,81]. Constitutively active ALK fusion proteins promote tumorigenesis through the activation of multiple signal transduction pathways by phosphorylating and activating signaling transmitters and pathways, such as phospholipase C γ (PLC γ), phosphatidylinositol 3-kinase (PI3K)/serine threonine kinase-1 (AKT-1), signal transducer and activator of transcription 3 (STAT3) and signal transducer and activator of transcription 5 (STAT5), mechanistic target of rapamycin (mTOR), and mitogen-induced extracellular kinase (MEK)/extracellular signal-regulated kinase (ERK) [82,83,84,85,86]. STAT3 oncogenic activity is mediated by its regulation of different target genes involved in the cell cycle, apoptosis, immune response, angiogenesis, and metabolism [87]. One of the important roles of STAT3 in ALCL is mimicking physiological progrowth signals, such as IL2 and TCR signaling pathways, allowing for the proliferation and survival of tumor cells [88,89]. Recent studies have revealed interferon regulatory factor 4 (*IRF4*) as a STAT3 key target gene, promoting survival of the neoplastic cells by activation of the transcription factor MYC. However, independently of *IRF4* genetic alterations (translocations or overexpression), IRF4 has been shown to induce the survival of ALCL neoplastic cells [90,91]. In myeloma cells, this mechanism is described as an autoregulatory circuit, with IRF4 directly targeting MYC and at the same time IRF4 representing a direct target of MYC transactivation [92].

Other mechanisms involving MYC may also be active in ALCL, such as BATF3 upregulation mediated by the JAK-STAT signaling pathway, leading to MYC activation promoting cell proliferation and apoptosis [93]. Consequently, inhibition of MYC and IRF4 represents a promising and novel therapeutic target strategy to overcome resistance in ALCL [90,91].

In addition, gene expression profiling has been able to detect transcriptional targets directly activated by the different signaling pathways promoting the growth and survival of the neoplastic cells [82,83,84,85,86]. One central downstream target of ALK in ALCL is the transcription factor CCAAT/enhancer binding protein beta (C/EBPβ). *C/EBPβ* is an intronless gene involved in various cellular processes, including differentiation, proliferation, inflammatory response, and metabolism [94]. This transcription factor is overexpressed in ALK+ ALCL and its expression is dependent on ALK kinase activity, indicating a critical role in the proliferation and survival by transcriptional activation of its target genes, among them *BCL2A1*, *G0S2*, and *DDX21* [60,83].

Other oncogenic mechanisms of NPM-ALK have been reported [95], including favoring genome instability through phosphorylation of DNA mismatch repair proteins (MMR) [96]; inducing angiogenesis by upregulation of HIF1A and VEGFA [97,98]; promoting metastasis through reversion to stem cell-like phenotype with overexpression of SOX2, SALL4, and TWIST1 [99,100,101]; inducing tumor inflammation through upregulation of IL-21 [102]; evading the immune response favoring immunosuppression with overexpression of IL-10, TGFβ, and PD-L1 [103,104]; and resisting death through the upregulation of antiapoptotic genes *MCL-1* and *BCL2A1* [105,106,107].

Relatively little is known about secondary genetic alterations in ALK+ ALCL. Two secondary mechanisms have been recognized as being associated with ALK inhibitor resistance: the first one related to mutations in the *ALK* kinase domain impairing ALK protein binding; and the second as a consequence of secondary mutations in the MAPK signaling pathway, achieving ALK independence, resembling what has been reported for other ALK-expressing neoplasms [108,109].

Comparative genomic hybridization technologies (CGH) have demonstrated the presence of secondary chromosomal imbalances in 58% of ALK+ ALCL, including gains of 6q, 7p, 17p, and 17q24 and losses of 4q, 13-q21, and 11q14 [110].

### 2.5. Differential Diagnosis

Due to its unusual features, the differential diagnosis of systemic ALCL includes hematopoietic and nonhematopoietic neoplasms. One needs to be aware that ALCL can present some morphological and immunophenotypical characteristics mimicking metastatic carcinoma, such as sinusoidal growth pattern, loss of CD45 in half of the cases, EMA and focal cytokeratin expression, leading to a misdiagnosis of a nonhematopoietic neoplasm. The lack of diffuse cytokeratin positivity and the characteristic strong and uniform CD30 expression are key features in the differential diagnosis with nonhematopoietic neoplasms [45,117].

ALK+ ALCL should be distinguished from ALK+ large B-cell lymphomas and nonhematopoietic neoplasms expressing ALK such as inflammatory myofibroblastic tumors, rhabdomyosarcomas, and neuroblastomas [29,118,119]. ALK+ large B-cell lymphomas usually show a plasmablastic appearance with a similar sinusoidal growth pattern. The neoplastic cells lack CD20 and CD30 reactivity, but are positive for ALK, showing a granular cytoplasmic pattern indicative of the CTLC-ALK fusion protein [28]. In addition, rare cases of ALK+ MH have recently been described in children, with a sinusoidal infiltration pattern and phenotypically characterized by the expression of histiocytic markers [120].

## 3. Systemic ALK-Negative Anaplastic Large Cell Lymphoma (ALK− ALCL)

### 3.1. Definition and Clinical Features

Systemic ALK− ALCL has a similar morphology and phenotype to ALK+ ALCL, but by definition, lacks ALK rearrangement and ALK expression [1]. ALK− ALCL usually affects adults with a slight male predominance; the mean age of diagnosis is between 55 and 60 years [9]. Half of the cases involve lymph nodes, and only 20% of the cases show an extranodal presentation. Two thirds of the patients are diagnosed in stage III–IV of the disease and show an unfavorable prognosis, usually worse than in systemic ALK+ ALCL [10]. However, stratification of ALK+ and ALK− ALCL cases according to age and stage in some studies have demonstrated similar prognosis [121], and the recent detection of recurrent chromosomal translocations has allowed the stratification of ALK− ALCL into prognostic groups.

### 3.2. Morphological Features

The histological findings in ALK− ALCL recapitulate the classical features of ALK+ ALCL. Cases with lymph node involvement usually show effacement of the nodal architecture by solid, cohesive sheets of neoplastic cells. In some of the cases, the architecture is preserved and the neoplastic cells grow within the sinuses, mimicking a metastatic carcinoma. The neoplastic cells exhibit the same cytological features of ALK+ ALCL, including the frequent occurrence of hallmark cells. Some studies have pointed out differences in the cytological findings, including larger, more pleomorphic cells with sometimes multilobated nucleus and a higher nuclear-to-cytoplasm ratio [122]. In contrast to ALK+ ALCL, no morphological variants are recognized. Specifically, the small cell pattern does not occur in ALK− ALCL, based on the lack of features to distinguish these cases from PTCL-NOS CD30^+^ [1].

### 3.3. Immunophenotype

**Figure 2:**
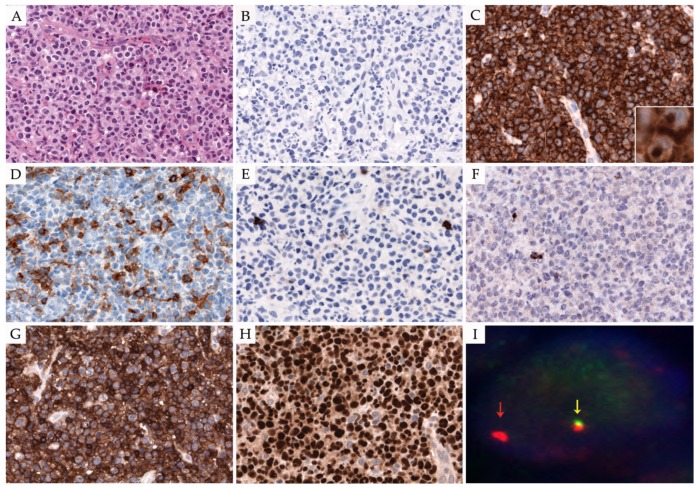
ALK− ALCL with DUSP-22 rearrangement. (**A**) Classic ALCL morphology is displayed; cells are relatively monomorphic (H&E stain, 400×); (**B**) Neoplastic cells lack ALK expression; (**C**) CD30 staining is strong and homogenous, showing a membranous and Golgi zone pattern (immunohistochemistry 400× and insert 630×); (**D**) CD4, (**E**) CD8; and (**F**) TIA-1 are negative in the neoplastic cells, exhibiting a triple-negative phenotype; (**G**) Neoplastic cells stained positive for TCR alpha-beta (beta F1), demonstrating the T-cell origin; (**H**) IRF4/MUM1 reveals nuclear expression in all neoplastic cells ((**B**,**D**–**H**); immunohistochemistry, 400×); (**I**) Fluorescent in situ hybridization (FISH) using a break-apart probe to the IRF4-DUSP22 locus on 6p25.3 shows one normal fusion signal (yellow arrow), one red signal (red arrow), and loss of one green signal, indicative of a IRF4-DUSP22 rearrangement. Abbreviations: H&E: hematoxylin and eosin.

ALK− ALCL, by definition, shows characteristically strong and homogenous CD30 expression. Pan-T-cell antigens such as CD3 are more commonly expressed in ALK− than in ALK+ ALCL cells. CD2 and CD3 are expressed in approximately 45% and 68%, respectively, whereas CD5 is often lost. CD4 is frequently expressed in the neoplastic cells and CD8 positivity is rare [65]. Although the neoplastic cells show a T-cell helper phenotype, cytotoxic markers such as TIA-1, granzyme B, and perforin are present, with the exception of cases with *DUSP22* rearrangement (see below, Figure 2) [122]. EMA immunostaining in ALK− ALCL is more variable as opposed to ALK+ ALCL, where most of the cases are positive [9]. PAX-5 and CD15 are useful markers for making a differential diagnosis with CHL. PAX-5 is usually weakly positive in RS cells and negative in ALK− ALCL; nevertheless, rare cases of ALK− ALCL with PAX-5 expression have been reported [38,123,124].

### 3.4. Genetic and Molecular Findings

The genetic driver alterations in ALK− ALCL were poorly understood until recently. New sequencing technologies have helped to identify new genetic alterations, cytogenetic abnormalities, and mutations in ALK− ALCL, and potential therapeutic targets. Cytogenetic abnormalities involving tyrosine kinase (*ROS1* and *TYK2*) with other gene partners than *ALK* have been found in a subset of cases, resulting in the activation of STAT3 or Janus kinase (JAK-1). The constitutive activation of the JAK-STAT signaling pathway as the central pathogenetic feature is shared with ALK+ ALCL. In addition to the cytogenetic abnormalities, whole exome sequencing has identified recurrent missense mutations affecting the SH2 domain of the *STAT3* gene (Y640F N647I, D661Y, and A662V) and the 1097 codon within the kinase domain of *JAK1* gene in 20% of the cases [102,125,126]. Although the frequency of STAT3 and JAK mutations is rather low (20%), STAT3 activation is seen in nearly half (47%) of ALK− ALCL cases [127]. Independently of JAK1/STAT3 mutational status, cases associated with STAT3 phosphorylation are sensitive to JAK1 inhibitors, presenting a promising therapeutic target [89].

Most importantly, two new distinctive chromosomal rearrangements in ALK− ALCL promoting its oncogenesis have been described. The first involves the *DUSP22-IRF4* locus (6p25.3), which is present in 30% of the cases; the second rearrangement involving *TP63* (3q28), a homolog of TP53, observed in 8% of the cases [128,129]. *DUSP22-IRF4* is rearranged frequently with the fragile site (*FRA7H*) on 7q32.3, resulting in a fusion protein, *DUSP22-FRA7H*, associated with the downregulation of dual specificity phosphatase gene (*DUSP22)* [128]. DUSP22 in normal T cells acts as a negative regulator of the TCR signaling by inactivating the MAPK-ERK2 pathway [130]. It is important to highlight the association of *DUSP22* rearrangements with a favorable outcome in ALK− ALCL, similar to ALK+ ALCL. In contrast, *TP63* rearrangements, which frequently occur with the *BL1XR1* partner gene as the product of an inv(3)(q26q28), are often associated with poor outcome. The *TP63* rearrangements lead to a fusion protein, which is homologous to the oncogenic protein Δnp63, a dominant-negative p63 isoform that inhibits the p53 pathway [122,128].

According to the presence or the absence of the cytogenetic abnormalities mentioned above, three different subgroups of ALK− ALCL have been characterized: *DUSP22*-rearranged ALCLs, *TP63*-rearranged ALCLs, and triple-negative ALCLs lacking *DUSP22*, *TP63*, and *ALK* [122]. Recently, a correlative morphological characterization of the different cytogenetic subgroups has been performed. *DUSP22*-rearranged cases show a classic pattern with relatively monomorphic features, more common hallmark cells, and frequent so-called doughnut cells with nuclear pseudoinclusions (Figure 2). The large pleomorphic cells commonly described in ALK− ALCL are less frequently observed in this subgroup [131]. *TP63*-rearranged ALCLs have a wider morphological spectrum than *DUSP22*-rearranged ALCL; nonetheless, these cases also present hallmark cells and lack large pleomorphic cells [128,132]. As mentioned above, *DUSP22*-rearranged cases are usually negative for cytotoxic markers and often show double negativity for CD4 and CD8. Of note, *DUSP22*- and *TP63*-rearrangements are not specific for systemic ALK− ALCL, and can be present in pC-ALCL and other subsets of PTCL; however, in the setting of systemic ALK− ALCL, detection of these translocations can be useful to predict the outcome of the disease and may influence therapeutic strategies [122].

Gene expression profiling has also identified a new genetic subgroup expressing ERBB4, observed in 24% of systemic ALK− ALCL. *ERBB4* is a member of the tyrosine kinase receptor ERBB family, which includes epidermal growth factor receptors *EGFR* (*ERBB1*), *HER-2* (*ERBB2*), *HER3* (*ERBB3*), and *HER4* (*ERBB4*). Interestingly, alterations in these genes in carcinomas have been linked to oncogenesis, and nowadays are therapeutic target genes. The aberrant ERBB4 overexpression is a unique feature of systemic ALK− ALCL that needs to be explored further and may have potential as a target for specific therapies [113]. Secondary chromosomal imbalances are more frequent in ALK− ALCL than in ALK+ ALCL, occurring in 65% of the cases. Gains of 1q and 6p21 and losses of 6q21 and 17p13 are characteristic of ALK− ALCL, whereas gains of chromosome 7p as well as 13q loss are seen in both ALK+ and ALK− ALCL [110].

### 3.5. Differential Diagnosis

The major differential diagnosis of ALK− ALCL is CD30-positive PTCL-NOS, in which cases with overlapping pathological features occur. PTCL-NOS differs from ALK− ALCL by a more variable cytology, more heterogeneous and usually weaker expression of CD30, and more common positivity for CD2 and CD3. Other useful markers are the absence of EMA and cytotoxic markers in most PTCL-NOS [9,65]. It is important to highlight that the diagnosis of ALK− ALCL should be reserved for cases with morphological and immunophenotypic findings closely resembling the classical pattern of ALK+ ALCL [1]. CHL rich in RS cells with less mixed inflammatory background can be misdiagnosed as ALK− ALCL, and PAX5 expression is a useful tool for this differential diagnosis [38]. Another entity to be considered in the differential diagnosis of ALCL is a subset of DLBCL showing CD30 positivity. CD30-positive DLBCL usually show a centroblastic appearance, but in some cases can display anaplastic morphology; the positivity of B-cell markers (CD20, CD79) in the neoplastic cells and the absence of ALK are crucial to establish the diagnosis [133,134].

Secondary involvement of ALK− ALCL in the skin has to be distinguished from primary cutaneous CD30^+^ T-cell lymphoproliferative disorders including lymphomatoid papulosis (LyP), pC-ALCL, and borderline lesions. The differential diagnosis between these entities is difficult, and a comprehensive approach including clinical, histological, and immunophenotypic features is required to resolve these cases [135]. Of note, primary cutaneous CD30^+^ lymphoproliferative disorders may involve regional draining lymph nodes without meaning a systemic ALK− ALCL. Moreover, cases with regional lymph node involvement do not have a worse prognosis or overall survival.

## 4. Breast Implant-Associated Anaplastic Large Cell Lymphoma (BI-ALCL)

### 4.1. Definition and Clinical Features

**Figure 3:**
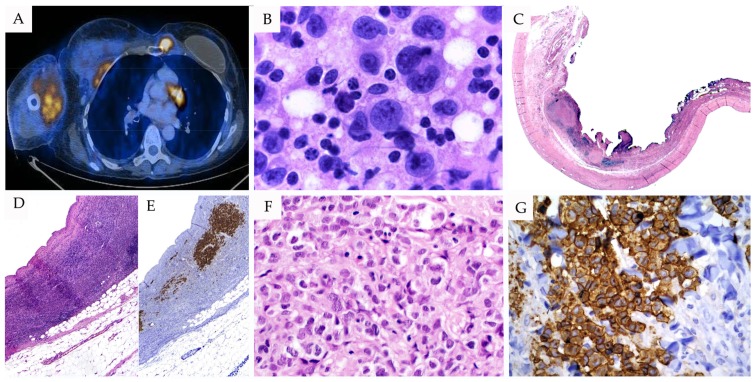
Breast implant-associated ALCL with systemic involvement. (**A**) 18 F-fluorodeoxyglucose-positron emission/computerized tomography (FDG-PET/CT) scan demonstrating hypermetabolic activity in the anterior right thorax and upper arm soft tissues accompanied by muscle and cutaneous involvement, maximum standardized uptake value-6 (SUV_max_-6). (**B**) Fine needle aspirate showing large neoplastic cells with abundant eosinophilic cytoplasm and eccentric horseshoe-shaped nuclei with multiple nucleoli (H&E stain, 630×). (**C**) Low-power view demonstrating capsule engrossment and extensive lymphoma infiltrate (H&E stain 25×). (**D**,**E**) Infiltration of the neoplastic cells to the surrounding soft tissue confirmed by CD30 immunostaining (H&E stain 50× and CD30 immunostaining 50×). (**F**) Sheets of large atypical neoplastic cells, as well as some occasional hallmark cells (H&E stain, 400×). (**G**) Neoplastic cells show strong and homogenous staining of CD30 (immunohistochemistry, 400×). Abbreviations: H&E: hematoxylin and eosin.

Breast implant-associated anaplastic large cell lymphoma (BI-ALCL) is a newly described CD30^+^ lymphoproliferation with characteristic clinical pathological findings that recently has been incorporated into the 2016 revised WHO classification as a provisional entity [1]. The first case was described by Keech and Creech in 1997, when they observed an association of ALCL with a saline-filled breast implant [136]. In 2011, the Food and Drug Administration announced a possible association between breast implants and the development of ALCL. Since then, 173 cases have been reported [137]. Even though many studies have shown a possible relationship between silicone breast implants and lymphoma, large epidemiological studies were not able to demonstrate an increased risk of lymphoma in patients with breast implants, indicating that the absolute risk is low [1]. All patients are women diagnosed between the ages of 24 and 82 years with history of breast implants, presenting with a mass or a periprosthetic fluid collection. According to the clinical presentation, two subgroups of this entity have been recognized. The first subgroup is the most frequent, clinically presenting as an effusion around the implant (also called seroma), whereas the second clinical subgroup presents with a palpable indolent tumor, which is generally confined to the breast, but occasionally can be associated with systemic involvement (Figure 3) [12,138,139].

Patients with BI-ALCL, in general, show an excellent outcome; however, some patients may develop recurrent disease associated with poor outcome. The reasons for these cases with poor prognosis remain unclear, and the association with systemic ALK− ALCL involving breasts with implants needs to be discussed [139,140]. In order to understand the pathogenesis and the natural history of this entity, further cohorts with longer clinical follow-up are warranted.

### 4.2. Morphological Features

Patients usually present with a seroma around the implant with liquid effusion ranging from 200 to 1000 mL, without gross evidence of tumor. The capsule is commonly thickened, showing a granular layer with adjacent fibrinoid material, but the implants usually are intact and not ruptured. The proliferation of the neoplastic cells is limited to the capsule and the effusion fluid, representing a type of in situ pattern. Cytologically, the tumor cells are large and pleomorphic, with abundant pale cytoplasm and vesicular, irregular-shaped nuclei. Some of these cells can exhibit a hallmark cell appearance. The background is polymorphic, consisting of small lymphocytes, histiocytes, and some granulocytes, mostly eosinophils [138,141]. The second subgroup of patients with a palpable mass shows a wider morphological spectrum. The neoplastic cells grow in a cohesive manner within a fibrotic or chronic inflammatory background and show multinodular appearance with geographic necrosis and abundant sclerosis (infiltrative pattern). Tumor cells are large with abundant cytoplasm and vesicular multilobated nuclei; typical hallmark cell morphology is rarely observed [138,141]. The histopathological findings in this entity are very characteristic and the differential diagnosis generally is not difficult. However, some cases show a predominant chronic inflammatory infiltrate composed by lymphocytes, histiocytes, and eosinophils, masking the neoplastic cells.

### 4.3. Immunophenotype

Neoplastic cells are strongly and uniformly positive for CD30 and usually negative for ALK (Figure 3) [138,139]. Rare cases have been reported showing ALK positivity [142,143]. CD43 is frequently positive in the neoplastic cells, but EMA shows a variable staining. T cell-associated antigens such as CD3, CD5, and CD7 are usually negative, but CD4 expression is present in most of the cases. CD8 is negative, but cytotoxic markers can be observed. CD45 and BCL2 are frequently positive, as well as IRF4/MUM1 [12,138]. BI-ALCL tumor cells, like their systemic counterparts, show phospho-STAT3 (p-STAT3) nuclear staining, demonstrating the constitutive activation of STAT3 and its role in the oncogenesis [12].

### 4.4. Genetic and Molecular Findings

TCR gene clonal rearrangements are present in the majority of cases [12,141]. Chromosomal abnormalities involving *ALK*, *DUSP22*, and *TP63* genes are absent, but recurrent mutations in the JAK-STAT3 pathway have been reported and can be related to p-STAT3 expression [12,116]. In addition, copy number variations showing gains of 19p and loss of chromosome 10p and 1p have been described; alterations that are not shared with systemic ALCL [116].

### 4.5. Differential Diagnosis

Breast implant history and anaplastic cell morphology is necessary for the diagnosis of BI-ALCL. Distinction between ALCL and other lymphomas such as CD30-positive PTCL and CHL involving the breast is necessary. Once the diagnosis of ALCL is established, the distinction between dissemination from systemic ALK− ALCL, pC-ALCL, and BI-ALCL is of crucial importance, due to their different clinical behaviors. These three entities share morphological and immunophenotypic features, and their distinction is frequently grounded in the clinical scenario [144].

## 5. Conclusions

ALCL encompasses a spectrum of mature T-cell malignancies sharing certain features such as anaplastic cytology, strong CD30 expression, and variable loss of T-cell markers, but displaying different clinical presentations, molecular features, and oncogenic mechanisms. Following the detection of ALK translocations as a defining feature and dominant oncogenic driver of ALK+ ALCL more than 20 years ago, the new genetic insights have now allowed the confirmation of ALK− ALCL as a distinct entity, setting it apart from other types of PTCL. The heterogeneity of ALCL is further underlined by the recognition of BI-ALCL, a new provisional entity with very specific clinico-pathological features associated with chronic inflammation. Further studies are needed to delineate the different oncogenic mechanisms of these lymphomas in order to better understand the different faces of ALCL and improve patient care.

## Figures and Tables

**Table 1:** Anaplastic large cell lymphoma (ALCL): classification and variants.

**1. Systemic ALK-positive ALCL (ALK+ ALCL)**
Morphological variants:
1.1. Common pattern 1.2. Small cell pattern 1.3. Lymphohistiocytic pattern 1.4. Hodgkin’s-like pattern 1.5. Composite pattern
**2. Systemic ALK-negative ALCL (ALK− ALCL)**
Genetic variants:
2.1. *DUSP-22-*rearranged ALCL 2.2. *TP63*-rearranged ALCL 2.3. Triple-negative ALCL 2.4. Others: *ERBB4*-aberrant expression
**3. Breast implant-associated ALCL (BI-ALCL)**
Clinical and morphological variants
3.1. Seroma (in situ pattern) 3.2. Palpable mass (infiltrative pattern), related to systemic involvement
**4. Primary cutaneous ALCL (pC-ALCL)**

**Table 2:** Immunophenotypic features of ALCL.

Marker	ALK+ ALCL	ALK− ALCL	BI-Associated ALCL
CD30	+	+	+
ALK	+	−	−
EMA	+	+	+
CD3	−/+	+/−	−/+
CD4	+/−	+/−	+/−
CD8	−	−/+	−/+
CD5	−/+	−/+	−/+
CD2	+/−	−/+	−/+
TCR BF1	−	−	N/A
TIA1	+/−	+/−	−/+
BCL6 ^1^	−/+	−/+	N/A
IRF4/MUM1	+	+	+/−

+: Positive in >90% of the cases; +/−: positive in >50% of the cases; −/+: positive in <50% of the cases; −: positive in <10% of the cases; N/A: not available [51,65,66]. ^1^ BCL6 is more frequently positive in ALK+ ALCL in comparison with ALK− ALCL (46% and 15%, respectively) [67].

**Table 3:** Genetic and molecular findings in ALCL.

Disease	Chromosomal Rearrangements	Gene Expression	Chromosomal Imbalances
ALK+ ALCL	Most common rearrangement (80%): NPM1-ALK, t(2;5)(p23;q35) [7] Other variant translocations: TPM3-ALK, t(1;2)(p23;q25) [72] ATIC-ALK, inv(2)(p23;q35) [75] TFG-ALK, t(2;3)(p23;q12) [71] CTLC-ALK, t(2;17)(p23;q23) [49,111] MSN-ALK, t(2;X)(p23;q11–12) [50] TPM4-ALK, t(2;19)(p23;p13) [69] MYH9-ALK, t(2;22)(p23;q11) [48] RNF213-ALK, t(2;17)(p23;q25) [69] TRAF-1-ALK, t(2;9)(p23;q33) [70]	*BCL-6*, *PTNP12*, *SERPINA1*, *CEBPB* [59] *STAT3* regulators: *IL-6*, *IL3IRA* [112]	Present in 58% of the cases: Gains of 6q, 7p, 17p, and 17q24 Losses of 4q, 13-q21, and 11q14 [110]
ALK− ALCL	IRF4-DUSP-22, 6p25.3 (30%) TP63-TBL1RXR1, 3q28 (8%) *JAK-1* and *STAT3* mutations variants resulting in STAT3 or JAK-1 constitutive activation: NKB2, ROS1 NCOR2-ROS1 NFKB2-TYK2 PABPC4-TYK2	*ERBB4* overexpression (24% of the cases) [113] *TNFSRF8*, *BATF*, *TMOD1*, *CCR7*, *CNTFR*, *IL22*, and *IL21* [59,114]	Present in 65% of the cases: Gains of 1q, 6p21, and 7p Losses of 6q21 (PRDM1), 13q, and 17p13 (TP53) [110,115]
BI-ALCL	No chromosomal translocations have been described to date	Not known	Gains of 19p Losses of 10p and 1p [116]
